# Supplementary material for: Lignocellulose-mediated selection of potential halophilic PET-degrading enzymes from mangrove soil
Source: Nat Commun. 2026 Apr 7;17:4930. doi: 10.1038/s41467-026-71548-z (PMC13234336; doi:10.1038/s41467-026-71548-z)
Supplement: Supplementary file 1 — Supplementary Information [file 41467_2026_71548_MOESM1_ESM.pdf]

**Lignocellulose-mediated selection of potential halophilic PET-degrading enzymes from mangrove soil**

María Fernanda Peña-Valencia [1 $], Semidán Robaina-Estévez [2], Gordon F. Custer [3], Onur Turak [4], Felipe Sierra [1], Lucas William Mendes [5], Carolina Rubiano-Labrador [6], Jay Gutiérrez [2], Annika Vaksmaa [7], Francisco Dini-Andreote [8,9], Alexandre Soares Rosado [10 *], Alejandro Reyes [1 *], Diego Javier Jiménez [10 $ *]

1. Department of Biological Sciences, Universidad de los Andes, Bogotá, Colombia
2. NewAtlantis Labs, Inc., City of Wilmington, Delaware, USA
3. Department of Natural Sciences, The University of Maryland Eastern Shore, Princess Anne, MD, USA
4. Department of Biochemistry, University of Bayreuth, Bayreuth, Germany
5. Cell and Molecular Biology Laboratory, Center for Nuclear Energy in Agriculture, University of São Paulo, Piracicaba, SP, Brazil
6. Chemical and Biological Studies Group, Basic Sciences Faculty, Universidad Tecnológica de Bolívar, Cartagena de Indias, Colombia
7. Department of Marine Microbiology and Biogeochemistry, Royal Netherlands Institute for Sea Research (NIOZ), The Netherlands
8. Department of Plant Science and Huck Institutes of the Life Sciences, The Pennsylvania State University, University Park, PA, USA
9. One Health Microbiome Center, The Pennsylvania State University, University Park, PA, USA.
10. Biological and Environmental Sciences and Engineering Division (BESE), King Abdullah University of Science and Technology (KAUST), Thuwal, 23955-6900, Kingdom of Saudi Arabia

$ These authors contributed equally: María Fernanda Peña-Valencia, Diego Javier Jiménez

* Corresponding authors: Alexandre Soares Rosado (alexandre.rosado@kaust.edu.sa); Alejandro Reyes (a.reyes@uniandes.edu.co); Diego Javier Jiménez (diego.jimenezavella@kaust.edu.sa)

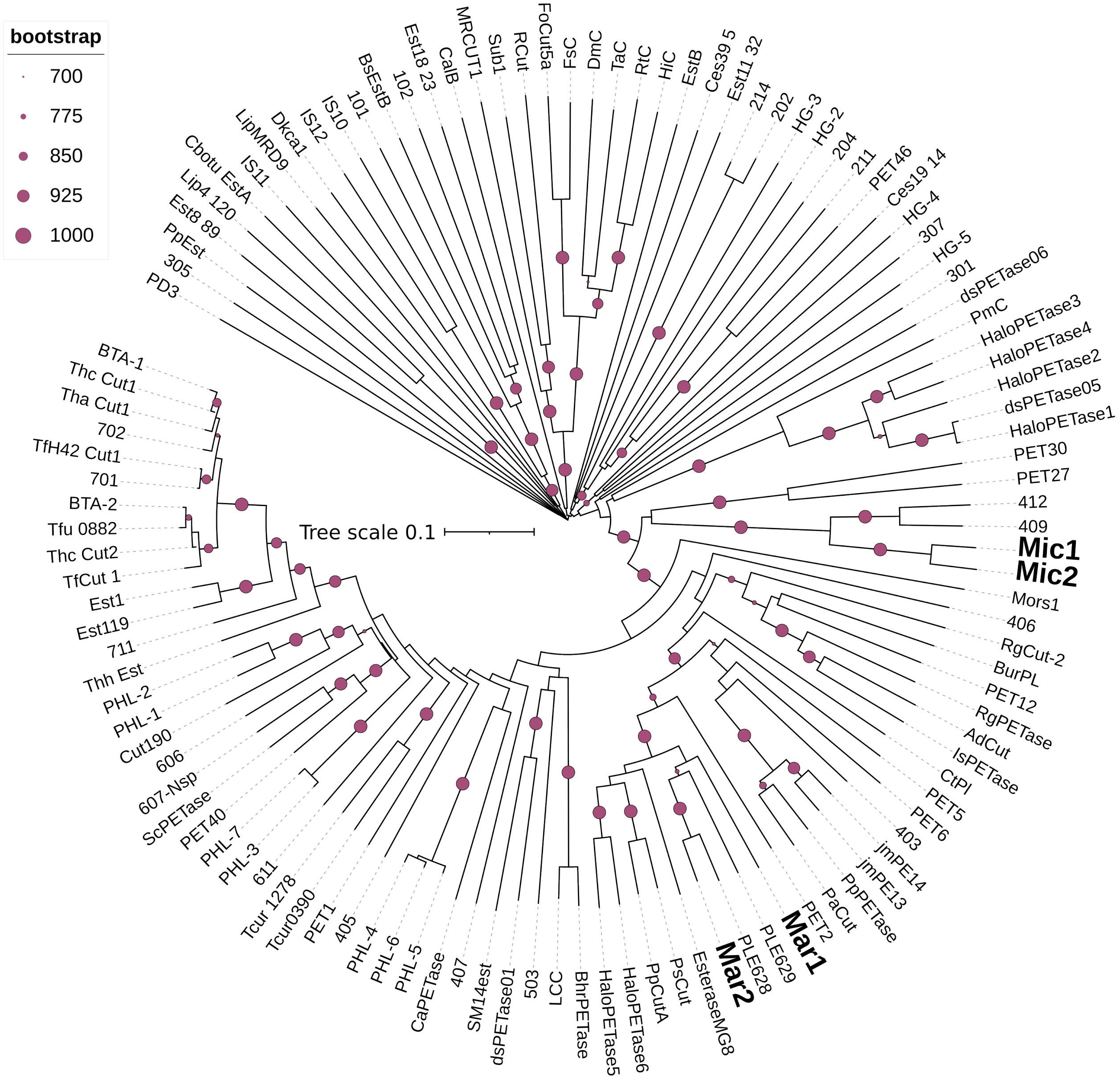


**Supplementary Fig. 1. Sequence placement of Mic1, Mic2, Mar1 and Mar2 in PETase landscape.** The reference sequences were inferred from the PAZy database. Neighbor-Joining tree NJ-tree was performed using multiple sequence alignment by ClustalX (v2.1) and in total 1000 bootstrap values. Labels of here-identified candidate PETases were highlighted boldly.
