# Supplementary material for: Lignocellulose-mediated selection of potential halophilic PET-degrading enzymes from mangrove soil
Source: Nat Commun. 2026 Apr 7;17:4930. doi: 10.1038/s41467-026-71548-z (PMC13234336; doi:10.1038/s41467-026-71548-z)
Supplement: Supplementary file 2 — Description of Additional Supplementary Files [file 41467_2026_71548_MOESM2_ESM.pdf]

Supplementary Data legends

Supplementary Data 1. Details of co-occurrence analysis of bacterial and fungal ASVs. Tabs in Excel file denoted S, C, CW, L and LW contain SparCC correlations used to create the network visualizations in Figure 2D.

Supplementary Data 2. Summary of metagenomic data processing steps.

Supplementary Data 3. Features of MAGs obtained in this study.

Supplementary Data 4. Gene annotations of MAGs based on COG functional categories.

Supplementary Data 5. Gene annotations of MAGs using CAZy families. Enrichment was assessed using over-representation analysis (ORA) based on hypergeometric tests applied to CDS-derived functional annotations, with p-values adjusted for multiple comparisons using Bonferroni correction; adjusted $p < 0.05$ was considered significant.

Supplementary Data 6. Gene annotations of MAGs using KEGG orthologs (KOs) relevant to lignin-transforming enzymes. Enrichment was assessed using over-representation analysis (ORA) based on hypergeometric tests applied to CDS-derived functional annotations, with p-values adjusted for multiple comparisons using Bonferroni correction; adjusted $p < 0.05$ was considered significant.

Supplementary Data 7. Gene annotations of MAGs using PAZy-derived enzymes.

Supplementary Data 8. FASTA files of putative PETases detected in this study, along with wild-type and known PET-active enzymes used for comparative analyses.

Supplementary Data 9. BLASTp output file of PETases containing the functional M5 motif

Supplementary Data 10. AI-based predictions of biophysical properties for putative PETases.

Supplementary Data 11. Detection of catalytic domains, active sites, and disulfide bonds in putative PETases.
