# Supplementary material for: Lignocellulose-mediated selection of potential halophilic PET-degrading enzymes from mangrove soil
Source: Nat Commun. 2026 Apr 7;17:4930. doi: 10.1038/s41467-026-71548-z (PMC13234336; doi:10.1038/s41467-026-71548-z)
Supplement: Supplementary file 10 — Supplementary Data 8 [file 41467_2026_71548_MOESM10_ESM.docx]

>403

MTTPTPTPEPEPEPPGGCGDCYQRGPDPTVAALEADRGPYSVRTINVSSWVSGFGGGTIHYPVGTQGTMGAIAVIPGYVSYENSIEWWGGRLASWGFVVITIDTNSIYDQPDSRANQLSAALDYVIAQSNSSRSAIQGMVDPNRLGAIGWSMGGGGTLKLSTDRYLKAAIPQAPWYSGFNPFDEITTPTLIIACQLDAVAPVAQHASPFYNEIPNSTAKAFLEIRNGDHFCANSGYPDEDILGKYGVAWMKRFIDDDRRYDAFLCGPNHEAEWDISEYRDTCNYLEHHHHHH

>503

MESPYERGPDPTSASVLDNGTFSLSSTSVSSLVTGFGGGTIYYPTSTTQGTFGGVVLAPGYTASSSSYSSVARRVASHGFVVFAIDTNSRYDQPDSRGSQILAAVSYLKNSASSTVASRLDETRIAVSGHSMGGGGTLAAANQDSSIKAAVALQPWHTDKTWPGIQIPTMIIGAENDSVAPVASHSIPFYTSMTGAREKAYGEINNGDHFIANTDDDWQGRLFVTWLKRYVDDDTRYSQFLCPAPSSIYLSDYRNTCPDLEHHHHHH

>606

MSNPYERGPAPTESSVTAVRGYFDTDTDTVSSLVSGFGGGTIYYPTDTSEGTFGGVVIAPGYTASQSSMAWMGHRIASQGFVVFTIDTITRYDQPDSRGRQIEAALDYLVEDSDVADRVDGNRLAVMGHSMGGGGTLAAAENRPELRAAIPLTPWHLQKNWSDVEVPTMIIGAENDTVASVRTHSIPFYESLDEDLERAYLELDGASHFAPNISNTVIAKYSISWLKRFVDEDERYEQFLCPPPDTGLFSDFSDYRDSCPHTTLEHHHHHH

>701

MANPYERGPNPTDALLEARSGPFSVSEENVSRLSASGFGGGTIYYPRENNTYGAVAISPGYTGTEASIAWLGKRIASHGFVVITIDTITTLDQPDSRAEQLNAALNHMINRASSTVRSRIDSSRLAVMGHSMGGGGSLRLASQRPDLKAAIPLTPWHLNKNWSSVRVPTLIIGADLDTIAPVLTHARPFYNSLPTSISKAYLELDGATHFAPNIPNKIIGKYSVAWLKRFVDNDTRYTQFLCPGPRDGLFGEVEEYRSTCPFLEHHHHHH

>IsPETase

MNFPRASRLMQAAVLGGLMAVSAAATAQTNPYARGPNPTAASLEASAGPFTVRSFTVSRPSGYGAGTVYYPTNAGGTVGAIAIVPGYTARQSSIKWWGPRLASHGFVVITIDTNSTLDQPSSRSSQQMAALRQVASLNGTSSSPIYGKVDTARMGVMGWSMGGGGSLISAANNPSLKAAAPQAPWDSSTNFSSVTVPTLIFACENDSIAPVNSSALPIYDSMSRNAKQFLEINGGSHSCANSGNSNQALIGKKGVAWMKRFMDNDTRYSTFACENPNSTRVSDFRTANCSLEHHHHHH

>LCC

MSNPYQRGPNPTRSALTADGPFSVATYTVSRLSVSGFGGGVIYYPTGTSLTFGGIAMSPGYTADASSLAWLGRRLASHGFVVLVINTNSRFDYPDSRASQLSAALNYLRTSSPSAVRARLDANRLAVAGHSMGGGGTLRIAEQNPSLKAAVPLTPWHTDKTFNTSVPVLIVGAEADTVAPVSQHAIPFYQNLPSTTPKVYVELDNASHFAPNSNNAAISVYTISWMKLWVDNDTRYRQFLCNVNDPALSDFRTNNRHCQLEHHHHHH

>GlacPETase

MSVTSRALALGTALLLAHTAHAAPAPNVPGGERVCAYTSGLSSLSYASARVTYPCTLSKAAYPATTLTGGFSNTKEQMTWLSEHLSSHGYIVITITPRNIFGAPTGWESAHKAGIAKLRSERSRRASPLYNKLDPSKFALTGFSMGGGGALLAAADLGSQVKVAVPMAPFLGSNNPNYSAITAKVLIQAGANDTVANPSTVASYYQSLPTGISRALTTFRSASHLDWINTGNTNRQARLKTLVTSWLKVYLDGNSDYATYLDGAEHSRHLAEDWFTRFEYVR

>PHL7

MANPYERGPDPTESSIEAVRGPFAVAQTTVSRLQADGFGGGTIYYPTDTSQGTFGAVAISPGFTAGQESIAWLGPRIASQGFVVITIDTITRLDQPDSRGRQLQAALDHLRTNSVVRNRIDPNRMAVMGHSMGGGGALSAAANNTSLEAAIPLQGWHTRKNWSSVRTPTLVVGAQLDTIAPVSSHSEAFYNSLPSDLDKAYMELRGASHLVSNTPDTTTAKYSIAWLKRFVDDDLRYEQFLCPAPDDFAISEYRSTCPF

>T_halotolerans

MANPYERGPNPTNSSIEALRGPFRVDEERVSRLQARGFGGGTIYYPTDNNTFGAVAISPGYTGTQSSISWLGERLASHGFVVMTIDTNTTLDQPDSRASQLDAALDYMVEDSSYSVRNRIDSSRLAAMGHSMGGGGTLRLAERRPDLQAAIPLTPWHTDKTWGSVRVPTLIIGAENDTIASVRSHSEPFYNSLPGSLDKAYLELDGASHFAPNLSNTTIAKYSISWLKRFVDDDTRYTQFLCPGPSTGWGSDVEEYRSTCPF

>Halopseudomonas_bauzanensis

MINKNLSQSLLAMMAAGALLLSSSAFAVNPPTDGPTDPDQAYERGPDPSVAFLEAPTGPHSVRTSRVSGL

VSGFGGGTIHYPTGTTGTMAAIVVIPGFVSAESSIEWWGPKLASHGFVVMTIDTNTGFDQPPSRARQINN

ALDYLVSQNTSRTSPVNGMIDTERLGVIGWSMGGGGTLRVASEGRIKAAIPLAPWDTTRFRGVQAPTLIF

ACESDLIAPVRSHASPFYNQLPDDIDKAYVEINNGSHYCANGGGLNNDVLSRFGVSWMKRFLDNDTRYSQ

FLCGPNHESDRNISEYRGNCPY

>HaloPETase1

MKAPLFKLAALSLGVSLSSVALATNPGGGGGGSNPDTGTGFPGVSSFSADGSFATTSGSAGLSCTVFRPS

TLGANGLKHPIIVWGNGTTASPSTYSGILEHWASHGFVVIAANTSNAGTGQDMLNCVDYLTTQNNRSTGT

YANKLDLNRIGAAGHSQGGGGTIMAGQDYRIKVTAPFQPYTIGLGHNSSSQSNQNGPMFLMTGSADTIAS

PTLNALPVYNRANVPVFWGELSGASHFEPVGSAGDFRGPSTAWFRYHLMDDASAEDTFYGSNCDLCTDND

WEVRRKGIN

>HaloPETase5

MKQIKSNTLLSLFAASALLFSLSAAANNPAPTPTPTPTPGTPSAYQRGPAPSVSFLQASRGDLSVRTSRVSSFTSGFGGGTIHYPTGTTGTMAAIVVIPGYVSAESSIEWWGPKLASYGFVVMTIDTNSGFDQPPSRARQINSALDYLVSQNSSSRSPVRGMIDTNRLGVVGWSMGGGGTLRVAEEGRIKAAIPLAPWDTTNFRDNYTPTLIFACQSDVIAPVNQHASPFYNQIPSSTDKAFVELSGGSHYCGNGGGVYNNVLGRLGVSWMKRHLDQDTRYDQFLCGPNHESDSRISEYRGNCSN

>T_curvata

MSLRKSFGLLSATAALVAGLVAAPPAQAAANPYQRGPDPTESLLRAARGPFAVSEQSVSRLSVSGFGGGRIYYPTTTSQGTFGAIAISPGFTASWSSLAWLGPRLASHGFVVIGIETNTRLDQPDSRGRQLLAALDYLTQRSSVRNRVDASRLAVAGHSMGGGGTLEAAKSRTSLKAAIPIAPWNLDKTWPEVRTPTLIIGGELDSIAPVATHSIPFYNSLTNAREKAYLELNNASHFFPQFSNDTMAKFMISWMKRFIDDDTRYDQFLCPPPRAIGDISDYRDTCPHT

>L8_Streptomyces

MQRIRSAVTAVLAALVLGLALVPGATAAPAPAAYDVGEENVYRQWDFGGGTIYYPEGAGPFPAVAISPGYTASQTSMAWYGPALAERGIVAFTIDTLSRFDQPGSRGDQLLAALDHLTEDSDVRHLVDPGRLGVMGHSMGGGGALEAAADRPSLRAAVALTPWNLDKTWGEVGTPTLIIGAENDTVASVATHSIPFYNSLPGGLDKAYLELNNASHFAPNSQDDTIETYTVAWLKVFLEGDAGYTSELCPGPGYSWEVSDYRSTCPF

>L11_Myceligenerans

MQSRSTARPPRATRRRPGLAAALAAVAVGLGALAAAPAQAADNPYERGPNPTESSIEATRGYYSVAQDSVSSLAPGFGGGTIYYPTTRTDGTFGAVAVVPGYTASESTIAWLGARVASQGFVVFTIDTDTRLDQPAQRGDQLLAALDYMTERSDVADRIDPSRLAVMGHSMGGGGTLEAAKDRPSLKAIIPMTPWNLDKTWGEVEVPMLDFGAEYDTIASPATHAKPFYNSHGGEKMYIELNSATHFAPNSSNTTIAKYSIAWLKRWVDNDTRYTQFLCPTPTSWYYIEESESTCGF

>Micromonosopora_ERR12772901__k127_1301752_3

MRPTIMSRTRPVPGVVTRLCLTVALVAVAGVAAAAPAQAQTNPYQRGPAPTNAILEASRGPFATSQQSVSSLVGGFGGGVIYYPTTTSEGTFGAVAISPGYTASWSSLAWLGPRIASHGFVVIGIETESRFDQPGSRGRQLLAALDYLVDRSSVRTRIDPDRLAVAGHSMGGGGSLEAAVDRPSLRAAVPIAPWNTDKTWTTVAVPTVGGEYDTIAPVYSHAEPFYDSIPGWTEKAYLELNGEGHLFPQVPNTELAKQGVAWLKRFVDDDTRYTQFLCPGPSGLAIEEYRSTCPF

>Microbulbifer_ERR12772901__k127_721586_2

MMKRKLTKKLLPTLGMILGGIFASAAVQAQSCPSDAICRYEDTPGNYSDNGPYGYDSYTMPYLSTPGGATVYYPRNAEPPYSLLVFTPPYTGTQIMYRDWGPFFASHGIALVTMDSRTIYDSVDSRADQQQDVLDAMKDENTRYGSPLRGKLDTSRFGATGWSMGGGATWITSAEYGGLKTAMSFAGHNLTAVDSDSSGRNTRIPTILFNGSLDTTYLGGLGQSDGVYRNIPYGVPKLFYEASNSGHFDWGGPEDANRYVGQLALAFQKTYLDGDTRWAQFLDRPPFYVGEFEKANIP

>Streptomyces_ERR12772901__k127_1254225_2

MQHSTTGGTRRRITGLLASVTVALGLGVAAGPAAVAADNPFERGPDPSESSIEAYRGAYSVSEQSVSRWVSGFGGGTIHYPTTRSDGTFGAVVVSPGYTGTESSISWLGPRLASQGFVVLTMATNSVYDYPDSRARQLQAALDYLTSSSSGVADRSDPDRLALMGHSMGGGGTLRAVSENPDIKAAIPMTPWHTDKTWREVTTPTLIFGAENDTIAPHYSHAEPFYDSLPSSLEKVYLELDGASHFAPNYSNTTIAKYSISWLKRFVDDDTRYSQFLCPGPSFWDGDVQTYESTCDF

>Spirillospora_ERR12772901__k127_86259_3

MHPTPRRAVKALLAAALAAGALAVPQHAAVAADNPYERGPAPTEQSVTAVRGPFATSQALVPSLGVTGFGGGTVYYPTSTAEGTFGAVAVAPGFTADQTSMAWLGPRLASQGFVVFTIDTLTRLDQPSSRAEQLKAALDYLTQRSGVRSRIDASRLGVMGHSMGGGGTLEAAEDRPQLQAAIPLTPWDLRKDFSGVRVPTMVIGAQADTVAPVLSHSEPFYSSIPAASEKAYLELRGASHFAPNIANTTIAKFSISWLKRYIDNDTRYDRFLCPPPGADLDISEYRDTCPNS

>Micromonosopora_ERR12772908__k127_878592_2

MHPSFPESEDPAPAARHSAAPLSRDCAHPPARRSTHRTAHRIAHRTARPDRSRRRIRGVLAAATAALLAVAGLTLIPNASAAENPFERGPAPSASSLAAARGPFATSQVTVSSRDASGFGGGTIYFPTSGDEGTFGAVAVAPGFTASRSSMAWLGPRLASQGFIVFNIDTITRSDQPASRGRQLLAALDFLVERSPIRDRVDPDRLAVMGHSMGGGGALEAAESRPTLRAAIPLTPWNLNKNWSRVQVPTMVIGAERDTIASVRFHSEPFFDSLPSDLDKAYLELNNASHFAPNIPNATISSFSVAWLKRFVDDDTRYEQFLCPPPRPDGEISEYQDTCARAS

>Marinobacter_ERR12772908__k127_2792925_3

MFKNLANNPVRSLIAAGSLLLSASAFAAGGGGTDDGDNGCTSNCGYERGPDPTVSFLEASTGPYSVRTDNVSSLVGGFGGGTVHYPTGTSGTMAAVVVIPGFVSAESSIEWWGPKLASYGFVVMTIDTNSGFDQPPSRATQINNALDYLIEQNGSSSSPYSGMIDTSRLGVIGWSMGGGGTLRVAAEGRLKAAIPLAPWDTSSYRFRDITTPTLIFACESDVVAPVGSHADPFYEAIPDSTSKAFVEMNNGSHYCGNGGNSYNDELGRLGVSWMKRFLDEDQRYSQFLCGPDHESDYRISEYRGTCPY

>Micromonosopora_ERR12772908__k127_1276758_2

MHPSFPESEDPAPAARHSAHPLSRDSVHPPARRWAHRSARPDRSRRRVRGVLAAAAAALLAVAGLTLIPNASAAENPFERGPAPSASSVAAARGPFATSQVTVSSRDASGFGGGTIYFPTSGDEGTFGGVAVAPGFTASRSSMAWLGPRLASQGFVVFNIDTVTRSDQPASRGRQLLAALDFLVERSPVRDRVDPDRLAVMGHSMGGGGALEAAEARPTLRAAIPLTPWNLSKNWSQVQVPTMVIGAERDTIASVRFHSEPFFDSLPSDLDKAYLELNNASHFAPNIPNTTISSFSVAWLKRFVDDDTRYEQLLCPPPRPGGEISEYQDSCARAS

>Isoptericola_ERR12772908__k127_861695_14

MQHSTTGGTRRRITGLLASVTVALGLGVAAGPAAVAADNPFERGPDPSESSIEAYRGAYSVSEQSVSRWVSGFGGGTIHYPTTRSDGTFGAVVVSPGYTGTESSISWLGPRLASQGFVVLTMATNSVYDYPDSRARQLQAALDYLTSSSSGVADRIDPDRLALMGHSMGGGGTLRAVSENPDIKAAIPMTPWHTDKTWREVTTPTLIFGAENDTIAPHYSHAEPFYDSLPSSLEKVYLELDGASHFAPNYSNTTIAKYSISWLKRFVDDDARYSQFLCPGPSFWDGDVQTYESTCDF

>Isoptericola_ERR12779715__k127_2806838_3

MQHSTTGGTRRRITGLLASVTVALGLGVAAGPAAVAADNPFERGPDPSESSIEAYRGAYSVSEQSVSRWVSGFGGGTIHYPTTRSDGTFGAVVVSPGYTGTESSISWLGPRLASQGFVVLTMATNSVYDYPDSRARQLQAALDYLTSSSSGVADRIDPDRLALMGHSMGGGGTLRAVSENPDIKAAIPMTPWHTDKTWREVTTPTLIFGAENDTIAPHYSHAEPFYDSLPSSLEKVYLELDGASHFAPNYSNTTIAKYSISWLKRFVDDDTRYSQFLCPGPSFWDGDVQTYESTCDF

>Marinobacter_ERR12779715__k127_2664185_2

MIKKSVRSALSVIAAGSLLFSASAMAINPETNPNPPSGCQTDCGFERGPDPTERFLEASRGHLSVRTSRVSGLVSGFGGGTIHYPTGTTGTMGAVVVIPGFVSAESSIDWWGPKLASYGFVVMTIDTNTGFDQPPSRARQINAALDYLIEQNGSSGSPIRGMIDTDRLGVIGWSMGGGGTLRVASEGRLSAAIPLAPWDTSSFQFNDIRTPTMIFACGADVIAPVALHASPFYNAIPDSTPKAFVQIGLGTHYCGNGGGLYNDVLGRLGVSWMKVHMDKDARYNQFICGPRHESDFNISEYRGNCR

>Microbulbifer_ERR12779715__k127_1039666_2

MIMMIMKRKLTSALPALGMILFGIFTATASQAQTCPSDAICRYEDTPGSYSDNGPFDYDSYTMPAFSTPGGATVYYPTSAAPPYSLLVFTPPYTGTQIMYRDWGPFFASHGIVLVTMDSRTIYDSVDSRADQQQDVLDAMKDENTRYGSPLRGKLDTNRFGATGWSMGGGATWITSAEYSGLKTAMSFAGHNLTAVDSDSSGRNTYIPTILFNGALDTTYLGGLGQSDGVYRNIPYGVPKLFYEASNAGHFAWGGPEDANRYVGQLALAFQKTFLDGDTRWAQYLDRPPLYVATFEKANIP
