# Supplementary material for: Lignocellulose-mediated selection of potential halophilic PET-degrading enzymes from mangrove soil
Source: Nat Commun. 2026 Apr 7;17:4930. doi: 10.1038/s41467-026-71548-z (PMC13234336; doi:10.1038/s41467-026-71548-z)
Supplement: Supplementary file 14 — Reporting Summary [file 41467_2026_71548_MOESM14_ESM.pdf]

nature portfolio

Corresponding author(s): Diego Javier Jimenez

Last updated by author(s): Feb 18, 2026

Reporting Summary

Nature Portfolio wishes to improve the reproducibility of the work that we publish. This form provides structure for consistency and transparency in reporting. For further information on Nature Portfolio policies, see our Editorial Policies and the Editorial Policy Checklist.

Statistics

For all statistical analyses, confirm that the following items are present in the figure legend, table legend, main text, or Methods section.

n/a	Confirmed	
☐	☒	The exact sample size (*n*) for each experimental group/condition, given as a discrete number and unit of measurement
☐	☒	A statement on whether measurements were taken from distinct samples or whether the same sample was measured repeatedly
☐	☒	The statistical test(s) used AND whether they are one- or two-sided<br>*Only common tests should be described solely by name; describe more complex techniques in the Methods section.*
☒	☐	A description of all covariates tested
☐	☒	A description of any assumptions or corrections, such as tests of normality and adjustment for multiple comparisons
☐	☒	A full description of the statistical parameters including central tendency (e.g. means) or other basic estimates (e.g. regression coefficient) AND variation (e.g. standard deviation) or associated estimates of uncertainty (e.g. confidence intervals)
☐	☒	For null hypothesis testing, the test statistic (e.g. *F*, *t*, *r*) with confidence intervals, effect sizes, degrees of freedom and *P* value noted<br>*Give P values as exact values whenever suitable.*
☒	☐	For Bayesian analysis, information on the choice of priors and Markov chain Monte Carlo settings
☒	☐	For hierarchical and complex designs, identification of the appropriate level for tests and full reporting of outcomes
☒	☐	Estimates of effect sizes (e.g. Cohen's *d*, Pearson's *r*), indicating how they were calculated

Our web collection on statistics for biologists contains articles on many of the points above.

Software and code

Policy information about availability of computer code

Data collection	We did not use any software or code to collect data in this study
Data analysis	Raw 16S rRNA gene sequences were processed with the DADA2 pipeline v1.26. The processed sequences were imported into PhyloSeq (v1.48.0) for diversity analyses. ASVs associated with distinct treatment groups were then identified using the Boruta algorithm and DESeq2 v1.5.0.2. Nonrandom co-occurrence analyses were performed using SparCC. Taxonomic profiling of metagenomic reads was performed using Kraken2 v1.4.0, with Bracken v2.9. For each treatment, high-quality and clean reads were co-assembled into contigs using the de novo assembler MEGAHIT v1.1.3. Assembly quality was evaluated with QUAST v5.0.2. Clean reads were mapped to contigs using Bowtie2 v2.4.5, and contigs were binned with MaxBin2 v2.2.7, MetaBAT2 v2.15, and VAMB v4.1.3. Bin refinement across treatments was performed using DAS Tool v1.1.4, and bin quality was assessed with CheckM v1.2.1. MAGs were taxonomically classified using GTDB-Tk v1.4.0. Relative abundance of MAGs across samples was estimated with CoverM v0.7.0 (https://github.com/wwood/CoverM). For each MAG, protein-coding sequences (CDSs) were predicted using the DFAST pipeline v1.2.6. To assess the potential for plastic degradation, predicted CDSs were aligned against the PAZy database using DIAMOND v0.9. Custom scripts for processing, analysis, and visualization of metagenomic data are available at: https://github.com/mariafpv/LignoMangrove-MAGs or https://doi.org/10.5281/zenodo.18651101. To generate a comprehensive protein catalog from the microcosm-derived metagenomes, the open-source software VEBA was used. Code for the structural and characterization of putative PETases is available at: https://github.com/Robaina/Mangrove-PETases orhttps://doi.org/10.5281/zenodo.18656903. Enzyme biophysical properties were ranked using the ProtScout Python package (https://github.com/new-atlantis-labs/ProtScout).

For manuscripts utilizing custom algorithms or software that are central to the research but not yet described in published literature, software must be made available to editors and reviewers. We strongly encourage code deposition in a community repository (e.g. GitHub). See the Nature Portfolio guidelines for submitting code & software for further information.

Data

Policy information about availability of data

All manuscripts must include a data availability statement. This statement should provide the following information, where applicable:

- Accession codes, unique identifiers, or web links for publicly available datasets
- A description of any restrictions on data availability
- For clinical datasets or third party data, please ensure that the statement adheres to our policy

Raw sequencing data are available through the European Nucleotide Archive (ENA) under BioProject ID PRJEB72453 (https://www.ebi.ac.uk/ena/browser/view/PRJEB72453). Source data are provided with this paper. Additional data generated in this study are provided in the Supplementary Information/Source Data file.

Research involving human participants, their data, or biological material

Policy information about studies with human participants or human data. See also policy information about sex, gender (identity/presentation), and sexual orientation and race, ethnicity and racism.

Reporting on sex and gender	NA
Reporting on race, ethnicity, or other socially relevant groupings	NA
Population characteristics	NA
Recruitment	NA
Ethics oversight	NA

Note that full information on the approval of the study protocol must also be provided in the manuscript.

Field-specific reporting

Please select the one below that is the best fit for your research. If you are not sure, read the appropriate sections before making your selection.

[x] Life sciences [ ] Behavioural & social sciences [ ] Ecological, evolutionary & environmental sciences

For a reference copy of the document with all sections, see nature.com/documents/nr-reporting-summary-flat.pdf

Life sciences study design

All studies must disclose on these points even when the disclosure is negative.

Sample size	A total of 25 soil samples (~500 g each) (n=25) were collected from the top 10 cm. To capture environmental heterogeneity, samples were taken at random intervals along a ~50 m transect extending inland from the shoreline. Then, soil samples were homogenized for subsequent microcosms experiments.
Data exclusions	Whole metagenome sequencing was performed only after 90 days of incubation (n=18), where desiccation was higher and soil samples were kept in longer time contact with the polymers.
Replication	All microcosm treatments (L, LW, P, PW, C, and CW) were replicated four times, resulting in a total of 48 experimental units (n = 48). The replication (4X) in each microcosm treatment was highly consistent. This was verified by PERMANOVA test on beta diversity analyses (Bray–Curtis distances).
Randomization	Mangrove soil samples were random taken and homogenized before start microcosms experiments (48 experimental units in three main treatments) in which randomization was not relevant because each biological replicate was used for microbiome analyses.
Blinding	Blinding was not relevant in this study due that researchers need to know the origin of samples to perform the correct analysis and needed comparisons. Negative controls (e.g. microcosm without polymers and original soil sample) were used during the experiments

Reporting for specific materials, systems and methods

We require information from authors about some types of materials, experimental systems and methods used in many studies. Here, indicate whether each material, system or method listed is relevant to your study. If you are not sure if a list item applies to your research, read the appropriate section before selecting a response.

Materials & experimental systems

n/a	Involved in the study
☒	☐ Antibodies
☒	☐ Eukaryotic cell lines
☒	☐ Palaeontology and archaeology
☒	☐ Animals and other organisms
☒	☐ Clinical data
☒	☐ Dual use research of concern
☒	☐ Plants

Methods

n/a	Involved in the study
☒	☐ ChIP-seq
☒	☐ Flow cytometry
☒	☐ MRI-based neuroimaging

Plants

Seed stocks	NA
Novel plant genotypes	NA
Authentication	NA
